# Double Nitrogenation Layer Formed Using Nitric Oxide for Enhancing Li^+^ Storage Performance, Cycling Stability, and Safety of Si Electrodes

**DOI:** 10.1002/advs.202310062

**Published:** 2024-04-24

**Authors:** Rahmandhika Firdauzha Hary Hernandha, Bharath Umesh, Jagabandhu Patra, Chung‐Jen Tseng, Chien‐Te Hsieh, Ju Li, Jeng‐Kuei Chang

**Affiliations:** ^1^ Department of Materials Science and Engineering National Yang Ming Chiao Tung University 1001 University Road Hsinchu 30010 Taiwan; ^2^ Hierarchical Green‐Energy Materials (Hi‐GEM) Research Center National Cheng Kung University 1 University Road Tainan 70101 Taiwan; ^3^ Department of Mechanical Engineering National Central University 300 Jhong‐Da Road Taoyuan 320317 Taiwan; ^4^ Department of Chemical Engineering and Materials Science Yuan Ze University 135 Yuandong Road Taoyuan 320315 Taiwan; ^5^ Department of Nuclear Science and Engineering and Department of Materials Science and Engineering Massachusetts Institute of Technology 77 Massachusetts Avenue Cambridge MA 02139 USA; ^6^ Department of Chemical Engineering Chung Yuan Christian University 200 Chung Pei Road Taoyuan 32023 Taiwan

**Keywords:** carbon coating, high energy density, high‐stability anode, nitrogenation, silicon nitride

## Abstract

To enhance Li storage properties, nitrogenation methods are developed for Si anodes. First, melamine, urea, and nitric oxide (NO) precursors are used to nitrogenize carbon‐coated Si particles. The properties of the obtained particles are compared. It is found that the NO process can maximize the graphitic nitrogen (N) content and electronic conductivity of a sample. In addition, optimized N functional groups and O─C species on the electrode surface increase electrolyte wettability. However, with a carbon barrier layer, NO hardly nitrogenizes the Si cores. Therefore, bare Si particles are reacted with NO. Core‐shell Si@amorphous SiN*_x_* particles are produced using a facile and scalable NO treatment route. The effects of the NO reaction time on the physicochemical properties and charge–discharge performance of the obtained materials are systematically examined. Finally, the Si@SiN*_x_* particles are coated with N‐doped carbon. Superior capacities of 2435 and 1280 mAh g^−1^ are achieved at 0.2 and 5 A g^−1^, respectively. After 300 cycles, 90% of the initial capacity is retained. In addition, differential scanning calorimetry data indicate that the multiple nitrogenation layers formed by NO significantly suppress electrode exothermic reactions during thermal runaway.

## Graphical abstract

A unique double nitrogenation layer on Si cores is developed. An electrode with this double nitrogenation layer shows significantly suppressed exothermic reactions during thermal runaway. A Si/NO/C‐N||LiNi_0.8_Co_0.1_Mn_0.1_O_2_ full cell exhibits a high energy density of 585 Wh kg^−1^ and retains >90% of its initial capacity after 300 cycles. The proposed double nitrogenation method is facile, cost‐effective, and scalable for practical applications.

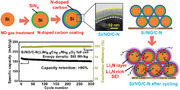

## 1 Introduction

Lithium‐ion batteries (LIBs) have become mainstream energy storage devices for a variety of applications, including portable electronic devices, electric vehicles, and grid‐scale energy storage systems.^[^ ^1^, ^2^, ^3^, ^4^ ^]^ However, the desired battery performance (e.g., that which would reduce the range anxiety of electric vehicle drivers) is beyond that of state‐of‐the‐art LIBs that use layered‐oxide positive electrodes and carbonaceous negative electrodes.^[^ ^5^ ^]^ The replacement of conventional graphitic electrodes, which have a practical capacity of ≈360 mAh g^−1^, with a higher‐capacity anode material is highly desired. In this context, Si‐based electrodes are of great interest because of their high theoretical capacity (≈3579 mAh g^−1^), appropriate lithiation/delithiation potential, high abundance (28% of Earth's crust by mass), low cost, and nontoxicity.^[^ ^6^, ^7^, ^8^ ^]^ However, Si anodes experience huge volume variation (>300%) during cycling, which causes mechanical disintegration of the electrodes, repeated solid‐electrolyte interphase (SEI) breakdown/formation, electrolyte consumption, and cyclable Li^+^ loss, limiting the electrode lifespan.^[^ ^9^ ^]^ Many approaches have been applied to overcome these problems, including electrode microstructure control,^[^ ^10^, ^11^, ^12^, ^13^ ^]^ electrode surface modification,^[^ ^14^, ^15^, ^16^ ^]^ binder engineering,^[^ ^17^, ^18^ ^]^ and electrolyte composition optimization.^[^ ^19^, ^20^, ^21^ ^]^ Numerous Si nanostructures have been developed to release the induced stress and strain during electrode cycling; however, their porosity (empty buffer space) decreases the electrode volumetric capacity and lowers the initial Coulombic efficiency (CE). A more practical and cost‐effective method for modifying Si anodes to increase mechanical/chemical stability and cyclability is highly desired.

Inorganic Si functional composites such as Si oxides/oxycarbides (e.g., SiO, SiO*_x_*, SiO_2_, SiOC),^[^ ^22^, ^23^ ^]^ Si carbide (SiC),^[^ ^24^, ^25^ ^]^ and Si nitrides (e.g., Si_3_N_4_, SiN*_x_*)^[^ ^26^, ^27^, ^28^ ^]^ have attracted increasing attention because these Si‐containing ceramic materials efficiently increase electrode cycling stability. Among them, Si nitrides have received a lot of research interest. Stoichiometric Si_3_N_4_ has excellent strength (>1 GPa) and high toughness (≈7.0 MPa m^1/2^).^[^ ^29^, ^30^ ^]^ It was reported that incorporated α‐Si_3_N_4_ serves as a framework to support active Si particles and facilitates Li^+^ transport within the electrode.^[^ ^31^, ^32^, ^33^ ^]^ In addition, Si_3_N_4_ prevents mechanical collapse and Si particle aggregation.^[^ ^26^, ^34^ ^]^ However, crystalline Si_3_N_4_ is relatively electrochemically inactive (specific capacity: ≈100 mAh g^−1^),^[^ ^34^ ^]^ which decreases the electrode specific capacity. The synthesis temperature for crystalline α‐Si_3_N_4_ is >1200 °C and that for crystalline β‐Si_3_N_4_ is >1400 °C, making fabrication energy‐consuming and necessitating the use of sophisticated equipment. Therefore, amorphous‐like SiN*_x_*, which has a high specific capacity of >1100 mAh g^−1^, is preferred. Generally, the synthesis temperature for SiN*_x_* is less than 1000 °C. The on‐site generated N‐containing SEI in the first charging process protects the SiN*_x_* electrode, leading to superior cyclability.^[^ ^33^ ^]^ The present study develops a facile and efficient route for nitrogenizing Si particles to create an amorphous‐like SiN*_x_* layer on Si cores.

Few studies have examined the creation of an amorphous‐like SiN*_x_* layer on Si particles. Paik et al. used ammonia (NH_3_) gas to treat Si nanotubes,^[^ ^35^ ^]^ forming an SiN/SiO*_x_* N*_y_* surface layer on the tubes. The obtained material showed a high initial capacity of 2131 mAh g^−1^ (CE: 86.3%) at 0.2 C. After 20 cycles, the electrode retained 96% of its initial capacity. Ng et al.^[^ ^33^ ^]^ and Huo et al.^[^ ^36^ ^]^ adopted a similar nitrogenation method to react a gaseous NH_3_ precursor with polycrystalline Si particles, producing amorphous SiN*_x_* shells on Si cores. The Si/SiN*_x_* composite anode had a decent capacity of 1400 mAh g^−1^ at 0.5 A g^−1^ after 200 cycles, which can be attributed to improved stress management and electrode conductivity owing to the nitrogenation layer.^[^ ^33^ ^]^ However, the NH_3_ precursor is considered hazardous and highly corrosive.^[^ ^37^, ^38^, ^39^ ^]^ Moreover, it is flammable and toxic and thus may have an environmental impact.^[^ ^40^ ^]^ Liu et al. synthesized a microscale N‐doped Si composite using an alternative method.^[^ ^41^ ^]^ The mechanical milling of sintered Si‐*x*LiNH_2_ mixtures under a CO_2_ atmosphere led to the in situ formation of an amorphous Li_2_CO_3_/SiO*_x_* shell (thickness: ≈10 nm), which contained dispersed SiN*_x_* clusters, on the Si particles. The resulting eggshell composite anode exhibited an initial reversible capacity of 1820 mAh g^−1^ at 0.1 A g^−1^ and a great rate capability of 1117 mAh g^−1^ at 2 A g^−1^.^[^ ^41^ ^]^ However, the LiNH_2_ precursor showed low nitrogenation capability and was sensitive to air and moisture (it thus requires an inert atmosphere^[^ ^42^ ^]^). The air‐tight ball‐milling process requires a delicate chamber design and is thus impractical for large‐scale production. A better precursor and a more scalable nitrogenation strategy for Si particles need to be developed.

In the present study, carbon‐coated Si particles are nitrogenized using various N‐containing precursors, namely melamine, urea, and nitric oxide (NO). Because bare Si particles show low electronic conductivity and poor cycling stability, carbon coating is commonly applied. Therefore, we adopt carbon‐coated Si particles as the starting material. The effects of precursor type on the physicochemical properties and charge–discharge performance of the obtained materials are systematically examined. It is found that the surface carbon layer can block the interaction between NO gas and Si particles. Therefore, we use NO to treat bare Si powder. The NO treatment time is varied to study its effect on electrode performance. The underlying mechanism of this effect is examined. Then, the NO‐nitrogenized Si particles are coated with N‐doped carbon to optimize the electrode charge–discharge properties. This is the first attempt to create a double nitrogenation layer on Si cores using NO gas. Because high‐energy‐density Si anodes can pose a high safety risk for batteries,^[^ ^43^ ^]^ we use differential scanning calorimetry (DSC) to evaluate the thermal properties of various nitrogenized Si samples after lithiation. The electrode with a double nitrogenation layer shows significantly suppressed exothermic reactions compared with those of the pristine Si electrode after the same lithiation process. The proposed nitrogenation method is facile, cost‐effective, and scalable for practical applications. Importantly, the rate capability, Li^+^ transport kinetics, redox transition reversibility, and cyclability of the electrode, which are crucial for next‐generation LIBs, are considerably enhanced.

## 2 Results and Discussion

**Figure 1:**
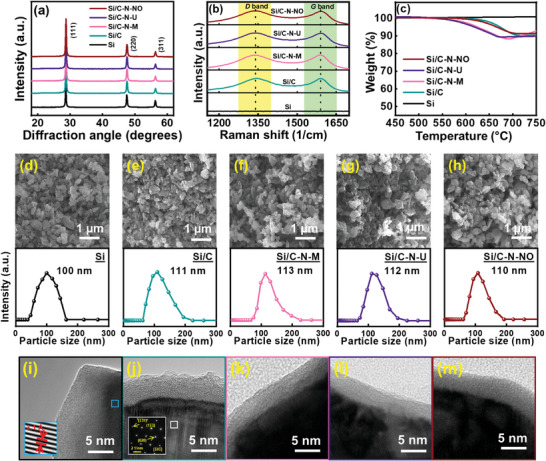
a) XRD patterns, b) Raman spectra, c) TGA curves of Si, Si/C, Si/C‐N‐M, Si/C‐N‐U, and Si/C‐N‐NO samples. SEM/DLS data for d) Si, e) Si/C, f) Si/C‐N‐M, g) Si/C‐N‐U, and h) Si/C‐N‐NO samples. High‐resolution TEM images for i) Si, j) Si/C, k) Si/C‐N‐M, l) Si/C‐N‐U, and m) Si/C‐N‐NO samples.

**Figure** **1**a shows the X‐ray diffraction (XRD) patterns of pristine Si, carbon‐coated Si particles (Si/C), and carbon‐coated Si particles nitrogenized using melamine (Si/C‐N‐M), urea (Si/C‐N‐U), and NO (Si/C‐N‐NO), respectively (see Section 4.1 for details). The peaks at 28.4°, 47.4°, and 56.1° are indexed as the (111), (220), and (311) plane diffractions, respectively, of cubic Si (JCPDS‐27‐1402). Due to the low crystallinity of the coating layers, no corresponding diffraction signals were detected. Figure 1b shows the Raman spectra of the samples. All the coated samples exhibit a *D*‐band signal at ≈1345 cm^−1^ and a *G*‐band signal at ≈1595 cm^−1^, which are associated with defective carbon bonding and the Raman‐allowed in‐plane vibration of sp^2^ carbon, respectively.^[^ ^44^ ^]^ The *D*‐to‐*G*‐band intensity ratios of Si/C, Si/C‐N‐M, Si/C‐N‐U, and Si/C‐N‐NO are 0.98, 1.02, 1.02, and 1.03, respectively, reflecting the increase in the disordering of the carbon atoms with the incorporation of nitrogen (N) heteroatoms. The quantity of the coating layers was evaluated using thermogravimetric analysis (TGA); the data are shown in Figure 1c. The samples with surface coating layers underwent a clear weight loss of approximately 10 wt% at around ≈550–700 °C, which is attributed to the burnout of the carbon‐based coating layers.^[^ ^45^ ^]^ Figure 1d–h shows the morphology and particle size distribution of various samples examined using scanning electron microscopy (SEM) and dynamic light scattering (DLS), respectively. Irregularly granular morphologies due to mechanical milling were observed for all samples. The *D*_50_ values for the pristine Si, Si/C, Si/C‐N‐M, Si/C‐N‐U, and Si/C‐N‐NO particles are 100, 111, 113, 112, and 110 nm, respectively. The diameter increase of the Si powder after the coating process confirms that a surface layer formed on the particles. Figure 1i–m shows high‐resolution transmission electron microscopy (TEM) images of various samples. While the Si cores are highly crystalline (as indicated by the lattice images and diffraction pattern shown in the figure), amorphous coating layers (thickness: ≈5 nm) were observed for the Si/C, Si/C‐N‐M, Si/C‐N‐U, and Si/C‐N‐NO samples. The N doping did not significantly alter the thickness and crystallinity of the coating layers. The tap densities of these samples were found to be approximately 0.28, 0.57, 0.64, 0.67, and 0.69 g cm^−3^, respectively (see Table S1, Supporting Information). The surface coating, especially with N doping, seems to reduce the electrostatic repulsion between Si particles, leading to an increase in tap density.

**Figure 2:**
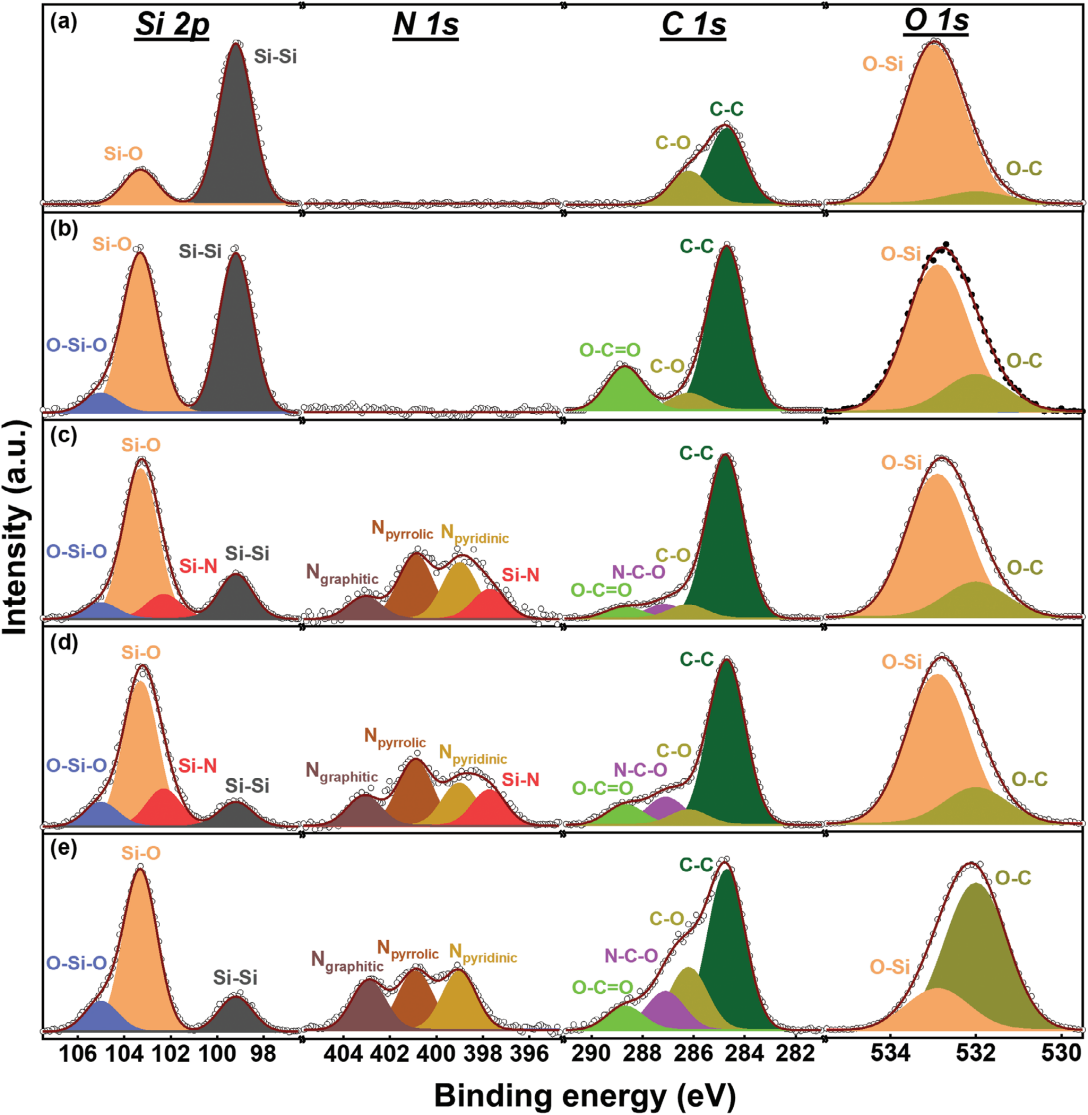
XPS data for a) Si, b) Si/C, c) Si/C‐N‐M, d) Si/C‐N‐U, and e) Si/C‐N‐NO samples.

The surface chemical composition of the samples was examined using X‐ray photoelectron spectroscopy (XPS). The results (see Table S2, Supporting Information) indicate that the N content of the Si/C‐N‐M, Si/C‐N‐U, and Si/C‐N‐NO samples is ≈3.8 at%. **Figure** **2** shows the high‐resolution XPS scans (Si 2p, N 1s, C 1s, and O 1s spectra) for various samples. As shown, the Si 2p spectra can be deconvoluted into several components. The peaks at 99.2, 102.3, 103.3, and 105.0 eV are associated with Si─Si, Si─N, Si─O, and O─Si─O species, respectively.^[^ ^32^, ^46^ ^]^ Interestingly, the Si─N signal appeared for the Si/C‐N‐M and Si/C‐N‐U samples, but not for the Si/C‐N‐NO sample. The N 1s spectra can be deconvoluted into four components. The peaks at 397.7, 399, 400.9, and 403.1 eV are associated with Si–N, pyridinic, pyrrolic, and graphitic N species, respectively. The existence of pyridinic, pyrrolic, and graphitic N species for Si/C‐N‐M, Si/C‐N‐U, and Si/C‐N‐NO confirms that the N atoms of the precursors diffused into the carbon layers. Again, there is no Si─N signal for Si/C‐N‐NO, reflecting that in the presence of a carbon barrier layer, NO hardly nitrogenized the Si core. However, Si/C‐N‐NO has a relatively high content of graphitic N, which is known to effectively increase the electronic conductivity of the carbon layer.^[^ ^47^, ^48^ ^]^ The Si/C‐N‐M and Si/C‐N‐U were prepared using a wet chemical method, where an ethanol solution containing glucose and a nitrogen precursor (either melamine or urea) was used. During the process, the nitrogen precursors react with Si particles to form Si─N species. The C 1s spectra can be deconvoluted into four components. In addition to the C─C peak at 284.7 eV, C─O, N─C─O, and O─C═O signals are found at 286.2, 287.1, and 288.7 eV, respectively.^[^ ^26^ ^]^ The N─C─O bonds of the Si/C‐N‐M, Si/C‐N‐U, and Si/C‐N‐NO samples confirm that N was doped into the surface carbon layers. Of note, a comparison of the Si/C‐N‐M and Si/C‐N‐U samples indicates that the latter has higher concentrations of Si─N (in Si 2p spectra) and N─C─O (in C 1s spectra) species. These results suggest that the urea precursor (vs melamine precursor) has a higher nitrogenation ability. The O 1s spectra can be deconvoluted into two components, namely O─C and O─Si peaks at 532.0 and 532.9 eV, respectively. The relatively high O─C content of the Si/C‐N‐NO sample can be attributed to the oxidizing power of the NO gas.

**Figure 3:**
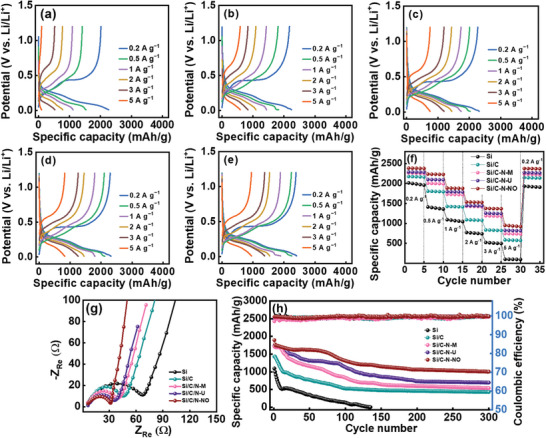
Charge‐discharge curves of a) Si, b) Si/C, c) Si/C‐N‐M, d) Si/C‐N‐U, and e) Si/C‐N‐NO electrodes measured at various rates. f) Comparative rate performance and g) EIS spectra of various electrodes. h) Cycling stability data for various electrodes measured at 1.0 A g^−1^.

Figure S1a–e (Supporting Information) shows the initial three charge–discharge curves of various electrodes measured at a current rate of 0.2 A g^−1^. The first‐cycle CE values of the pristine Si, Si/C, Si/C‐N‐M, Si/C‐N‐U, and Si/C‐N‐NO electrodes are 75%, 83%, 85%, 86%, and 87%, respectively. The efficiency loss is ascribed to SEI formation and the irreversible trapping of Li^+^ ions within the electrodes.^[^ ^49^ ^]^ Si/C‐N‐NO having the highest initial CE can be ascribed to it having the highest electronic conductivity among the electrodes (see Table S3, Supporting Information), which promotes redox reversibility. This could be associated with the relatively high graphitic N content of Si/C‐N‐NO (Figure 2e). The N_2_ adsorption/desorption data for Si/C and Si/C‐N‐NO are shown in Figure S2 (Supporting Information). Although the latter shows a higher Brunauer–Emmett–Teller surface area (80 vs 45 m^2^ g^−1^), its first‐cycle reversibility is better. **Figure** **3**a–e shows the charge–discharge profiles of the electrodes recorded at various current rates after two conditioning cycles. The reversible capacities obtained at 0.2 A g^−1^ are 2015, 2177, 2268, 2294, and 2389 mAh g^−1^ for the pristine Si, Si/C, Si/C‐N‐M, Si/C‐N‐U, and Si/C‐N‐NO electrodes, respectively. With increasing current rate, the specific capacities decreased, as shown in Figure 3f and **Table** **1**. The capacity values of these electrodes decreased to 101, 588, 749, 826, and 950 mAh g^−1^, respectively, at 5 A g^−1^, corresponding to 5%, 27%, 33%, 36%, and 40% of the capacities found at 0.2 A g^−1^. Besides electronic conductivity (Table S3, Supporting Information), electrolyte wettability is a crucial factor that affects the interface resistance and electrode rate capability. The contact angle measurement results shown in Figure S3 (Supporting Information) indicate that the N doping clearly increased the electrolyte wettability of the electrodes. The surface of the Si/C‐N‐NO electrode has an optimal N composition and high O─C content, and thus has superior wettability and may cause redox faradaic reactions.^[^ ^50^, ^51^ ^]^ As a result, the best charge–discharge properties, especially at a high rate, were obtained for the Si/C‐N‐NO electrode.

**Table 1:** Reversible specific capacities of pristine Si, Si/C, Si/C‐N‐M, Si/C‐N‐U, and Si/C‐N‐NO electrodes measured at various current rates.

Current rate [A g^−1^]	Pristine Si (mAh g^−1^]	Si/C [mAh g^−1^]	Si/C‐N‐M [mAh g^−1^]	Si/C‐N‐U [mAh g^−1^]	Si/C‐N‐NO [mAh g^−1^]
0.2	2015	2177	2268	2294	2389
0.5	1412	1809	2009	2101	2239
1	1093	1430	1732	1795	1888
2	773	1092	1433	1449	1535
3	521	837	1193	1260	1378
5	101	588	749	826	950
High rate retention^a)^	5%	27%	33%	36%	40%
Capacity retention after 300 cycles	0%	30%	32%	41%	53%

^a)^ a comparison between reversible capacities at 5.0 and 0.2 A g^−1^.

The causes of the rate capability difference between various electrodes are further examined using electrochemical impedance spectroscopy (EIS) and the galvanostatic intermittent titration technique (GITT). Figure 3g shows the EIS data of the electrodes acquired after two conditioning cycles. The Nyquist spectra consist of a semicircle at high frequency and a sloping line at low frequency, which can be characterized by the equivalent circuit shown in the figure inset, where *R*_e_, *R*_ct_, *CPE*, and *W* represent the electrolyte resistance, charge transfer resistance, interfacial constant‐phase element, and Warburg impedance associated with Li^+^ diffusion within the electrode, respectively.^[^ ^52^ ^]^ The *R*_ct_ values derived from the fitting results are 61, 39, 33, 29, and 25 Ω for the Si, Si/C, Si/C/N‐M, Si/C/N‐U, and Si/C/N‐NO electrodes, respectively. Figure S4 (Supporting Information) shows the GITT data of the electrodes measured during lithiation and delithiation. The apparent Li^+^ diffusion coefficients (*D*_Li_^+^) can be assessed based on these data (the calculation details are described in the note of Figure S4, Supporting Information).^[^ ^53^ ^]^ As shown in **Table** **2**, the Si/C/N‐NO electrode has the highest *D*_Li_^+^ values (i.e., 4.69 and 4.92 × 10^−10^ cm^2^ s^−1^ for lithiation and delithiation, respectively), followed by the Si/C/N‐U, Si/C/N‐M, Si/C, and pristine Si electrodes. The *R*_ct_ and *D*_Li_^+^ data reflect the charge transfer kinetics at the interface and within the bulk electrode, respectively, explaining the rate capability variation between the examined anodes.

**Table 2:** *R*_ct_ and *D*_Li_^+^ values of pristine various electrodes.

Electrode	*R*_ct_ after conditioning [Ω]	*R*_ct_ after 300 cycles [Ω]	Lithiation/delithiation D_Li_^+^ [× 10^−10^ cm^2^ s^−1^]
Si	61	116	1.25/1.84
Si/C	39	68	2.45/3.34
Si/C‐N‐M	33	50	3.30/3.75
Si/C‐N‐U	29	42	3.81/4.01
Si/C‐N‐NO	25	36	4.69/4.92
Si/NO‐0.5h	23	33	4.78/5.21
Si/NO‐1h	17	23	5.81/6.33
Si/NO‐2h	19	26	4.13/5.66
Si/NO/C‐N	15	19	6.72/7.04

Figure 3h shows the cycling stability data of the electrodes measured at 1.0 A g^−1^. The pristine Si showed almost no capacity after 130 cycles, whereas the Si/C, Si/C‐N‐M, Si/C‐N‐U, and Si/C‐N‐NO electrodes retained 30%, 32%, 41%, and 53% of their initial capacities, respectively, after 300 charge–discharge cycles. Figure S5a (Supporting Information) shows the EIS spectra of the electrodes after 300 charge–discharge cycles. As can be seen, the Nyquist circles evolve upon cycling (Figure 3g). The relatively small change in the *R*_ct_ values of the Si/C‐N‐NO electrode (Figure S5b, Supporting Information) suggests that the N‐doped carbon layer prepared using NO can stabilize the electrode/electrolyte interface, increasing cyclability.

**Figure 4:**
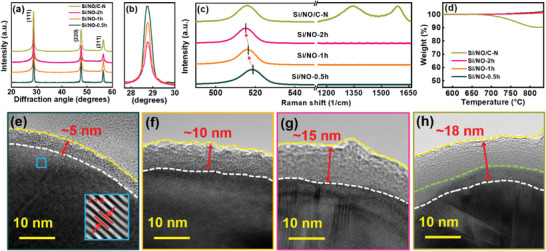
a) XRD patterns, b) Si (111) diffraction intensity, c) Raman spectra, and d) TGA curves for various samples. High‐resolution TEM images for e) Si/NO‐0.5 h, f) Si/NO‐1 h, g) Si/NO‐2 h, and h) Si/NO/C‐N samples.

The data above confirm that NO gas is a good precursor for doping the anode material. Moreover, NO at a concentration of only 1500 ppm is sufficient for inducing positive effects and the synthesis process is facile and scalable. However, with the carbon coating layer, nitrogenation hardly takes place in the Si cores. Therefore, in this section, we treat pristine bare Si with NO gas for various periods of time. The samples treated for 0.5, 1, and 2 h are denoted as Si/NO‐0.5 h, Si/NO‐1 h, and Si/NO‐2 h, respectively. Furthermore, we apply a glucose coating and NO heating to Si/NO‐1 h, producing Si/NO/C‐N with a double nitrogenation layer (i.e., an SiN*_x_* inner layer and an N‐doped carbon outer layer). **Figure** **4**a shows the XRD patterns of the samples. All the observed peaks are attributed to polycrystalline Si. No extra peaks appear after the NO treatment. However, as shown in Figure 4b, the Si diffraction peaks become broader and weaker with increasing NO treatment time, indicating the amorphization of the Si particles and the formation of an amorphous nitrogenation layer. Table S2 (Supporting Information) shows the XPS results, which reveal that the N content of the samples increases with increasing NO treatment time (i.e., 2.5 at% at 0.5 h and 5.0 at% at 2 h). As shown in the Raman spectra (Figure 4c), the peak related to the vibration band of polycrystalline Si^[^ ^46^ ^]^ shifts from 520 to 515 cm^−1^ with increasing NO treatment time. This is associated with Si lattice distortion and the formation of SiN*_x_* on the particles.^[^ ^41^ ^]^ Figure 4c also shows that carbon *D* and *G* bands appear for Si/NO/C‐N, confirming the existence of an N‐doped carbon coating on this sample. According to the TGA data in Figure 4d, the N‐doped carbon layer accounts for ≈10 wt% of the Si/NO/C‐N sample. The DLS data in Figure S6 (Supporting Information) show that the *D*_50_ values for the Si/NO‐0.5 h, Si/NO‐1 h, Si/NO‐2 h, and Si/NO/C‐N samples are 110, 118, 127, and 130 nm, respectively. A comparison with the *D*_50_ value of pristine Si (i.e., 100 nm) suggests that some reaction product layers formed after the NO treatment. Figure 4e–h shows high‐resolution TEM images of the prepared samples. As shown, the surface amorphous SiN*_x_* layer thickness increases from ≈5 to ≈15 nm when the NO treatment time is increased from 0.5 to 2 h. The Si/NO/C‐N sample shows a double nitrogenation layer (an inner SiN*_x_* layer and an outer N‐doped carbon layer). These two layers are well integrated, with intimate contact between them. Figure S7 (Supporting Information) shows low‐resolution TEM images of the Si/NO‐1 h and Si/NO/C‐N samples, demonstrating the integrity of the surface layers. Table S1 (Supporting Information) shows the tap densities of various samples. They indicate that the NO‐produced double nitrogenation layer can significantly consolidate the Si particles (0.28 g cm^−3^ for pristine Si and 0.84 g cm^−3^ for Si/NO/C‐N). A denser material is favorable for making a more compact electrode, which is beneficial for electrode volumetric performance.^[^ ^54^ ^]^

**Figure 5:**
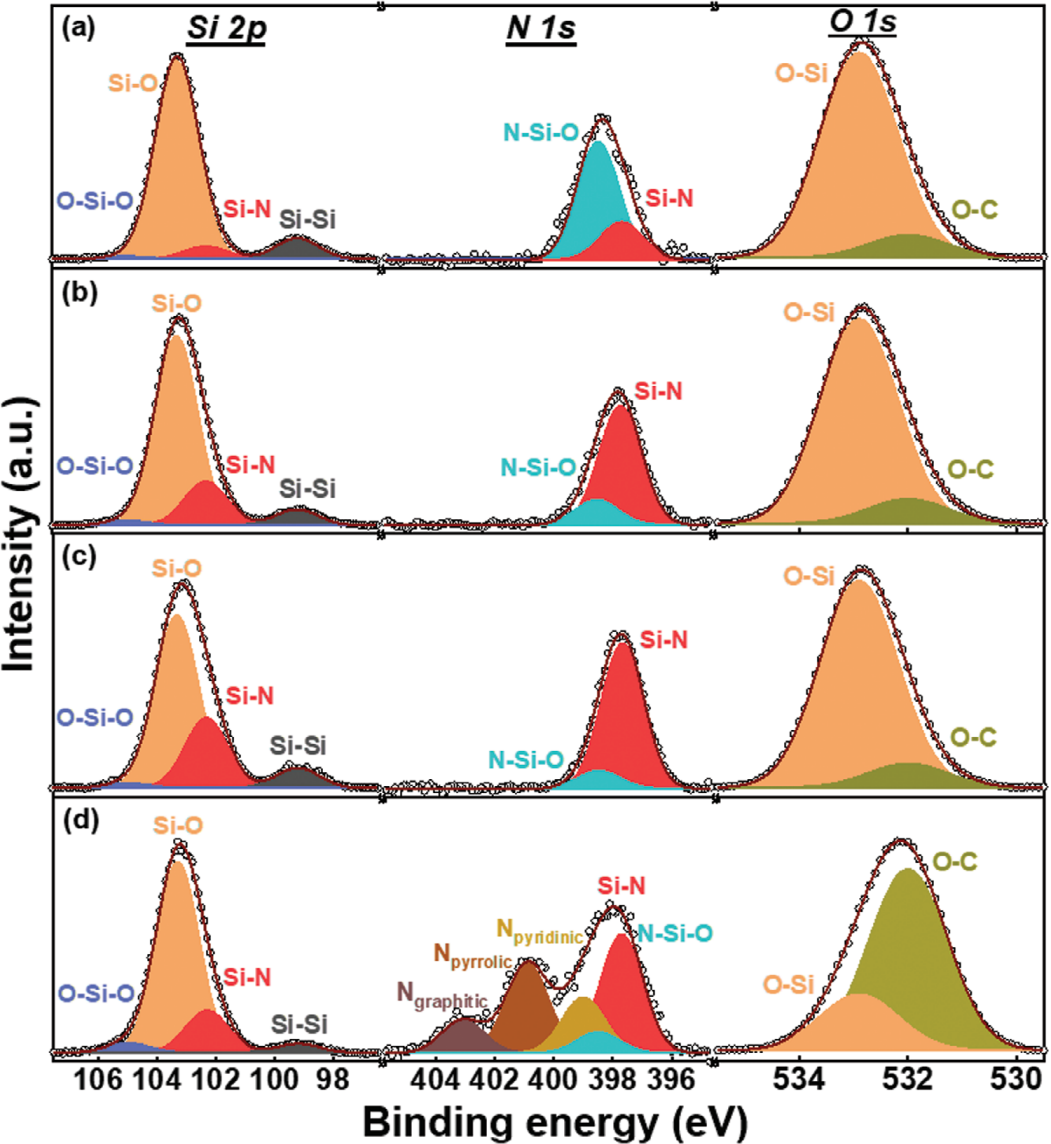
XPS data for a) Si/NO‐0.5 h, b) Si/NO‐1 h, c) Si/NO‐2 h, and d) Si/NO/C‐N samples.

**Figure** **5** shows the XPS spectra of the Si/NO‐0.5 h, Si/NO‐1 h, Si/NO‐2 h, and Si/NO/C‐N samples. The Si 2p spectra contain clear Si─N bonding signals, confirming that NO can effectively nitrogenize Si if the carbon barrier layer is absent. It is noted that the Si─N peak intensity increases with increasing NO treatment time. The N 1s spectra of the Si/NO‐0.5 h, Si/NO‐1 h, and Si/NO‐2 h samples show that with increasing NO treatment time, the Si─N signal overwhelms the N─Si─O signal. This indicates that the N atoms gradually diffused through the surface oxide layer to the Si cores. The N 1s spectrum of Si/NO/C‐N confirms successful N doping both in the carbon layer and in the Si matrix. As shown in the O 1s spectra, the Si─O component dominates for the samples without a surface coating, whereas the C─O component is the major species for the Si/NO/C‐N sample.

Figure S8a–c (Supporting Information) shows the initial charge–discharge curves of the Si/NO‐0.5 h, Si/NO‐1 h, and Si/NO‐2 h electrodes measured at 0.2 A g^−1^. The first‐cycle CE values are 81%, 85%, and 83%, respectively. The appropriate formation of Li_3_N, which is derived from the conversion reaction of SiN*_x_* during the first lithiation, can help passivate the electrode surface. However, an excessive irreversible conversion reaction, such as that for the Si/NO‐2 h electrode (due to its higher N content), could reduce the CE. Figure S8d–f (Supporting Information) shows the charge–discharge profiles of these three electrodes measured at various current rates after two conditioning cycles. Although the reversible capacities obtained at 0.2 A g^−1^ are similar for all electrodes, the capacities reduced to 1028, 1230, and 1151 mAh g^−1^, respectively, at 5 A g^−1^ (see **Table** **3**). Of note, these high‐rate properties are higher than those of the Si/C/N‐M, Si/C/N‐U, and Si/C/N‐NO electrodes shown in Table 1. The direct nitrogenation of bare Si particles seems to be more effective in improving electrode performance, probably because the created amorphous SiN*_x_* layer on the outermost particles could be more readily transformed to a Li_3_N‐based SEI, which is known to have high Li^+^ conductivity.^[^ ^55^ ^]^ According to the literature,^[^ ^55^, ^56^, ^57^, ^58^, ^59^, ^60^, ^61^, ^62^ ^]^ the Li^+^ conductivities of LiF, Li_2_CO_3_, Li_2_O, Li*_x_* SiO*_y_*, and Li_3_N are ≈10^−10^–10^−6^, ≈10^−8^, ≈10^−12^–10^−9^, ≈10^−7^–10^−5^, and ≈10^−4^–10^−2^ S cm^−1^, respectively. In addition, the higher tap density of the Si/NO samples (Table S1, Supporting Information) could facilitate Li^+^ and *e* ^−^ conduction among the particles, resulting in superior high‐rate performance. Figure S9a (Supporting Information) shows the EIS data of the electrodes acquired after two conditioning cycles. The *R*_ct_ values for the Si/NO‐0.5 h, Si/NO‐1 h, and Si/NO‐2 h electrodes are 23, 17, and 19 Ω, respectively (Table 2). The GITT data (see Figure S10, Supporting Information) indicate that the Si/NO‐1 h electrode has the highest *D*_Li_^+^ values for lithiation and delithiation, namely 5.81 and 6.33 × 10^−10^ cm^2^ s^−1^, respectively, among the three electrodes (Table 2). The increased *R*_ct_ and reduced *D*_Li_^+^ values for the Si/NO‐2 h electrode can be attributed to the excessive amount of Li_3_N formed, which increases both the Li^+^ transport distance across the SEI and electrode resistance. As shown in Figure S11 (Supporting Information), the SEI thickness of the Si/NO‐2 h electrode is clearly higher than that of the Si/NO‐1 h electrode.

**Table 3:** Reversible specific capacities of Si/NO‐0.5 h, Si/NO‐1 h, Si/NO‐2 h, and Si/NO/C‐N electrodes measured at various current rates.

Current rate [A g^−1^]	Si/NO‐0.5 h [mAh g^−1^]	Si/NO‐1 h [mAh g^−1^]	Si/NO‐2 h [mAh g^−1^]	Si/NO/C‐N [mAh g^−1^]
0.2	2390	2405	2397	2435
0.5	2240	2255	2247	2285
1	1889	1904	1896	1934
2	1689	1704	1696	1734
3	1532	1547	1539	1577
5	1028	1230	1151	1280
High rate retention^a)^	43%	51%	48%	53%
Cycle retention after 300 cycles	54%	77%	70%	90%

^a)^ a comparison between reversible capacities at 5.0 and 0.2 A g^−1^.

**Figure 6:**
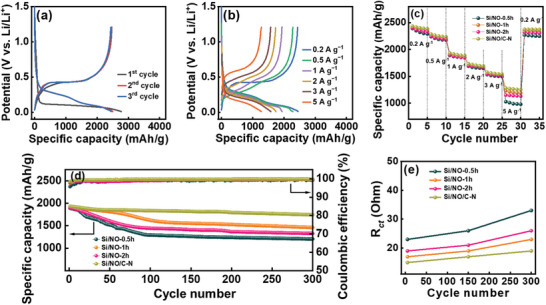
a,b) Charge–discharge curves of Si/NO/C‐N electrode recorded at 0.2 A g^−1^. c) Comparative rate performance of various electrodes. d) Cycling stability data for various electrodes measured at 1.0 A g^−1^. e) Variation of *R*_ct_ values for various electrodes with respect to charge–discharge cycle number.

Since an NO treatment time of 1 h is optimal for Si particles, we applied a glucose coating followed by NO heating to Si/NO‐1 h, producing Si/NO/C‐N. **Figure** **6**a shows that the first‐cycle CE of this electrode is as high as 89%, which can be ascribed to the good passivation of Li_3_N and high conductivity of the N‐doped carbon coating (see Table S3, Supporting Information). Moreover, the double nitrogenation layer minimized the mechanical breakdown of the particles, increasing electrochemical reversibility and thus the CE. As shown in Table S4 (Supporting Information), the initial CE of 89% is among the best values reported in the literature. A high first‐cycle CE is crucial for Si‐based anodes and determines their practical applicability.^[^ ^63^ ^]^ As shown in Figure 6b, the reversible capacities of the Si/NO/C‐N electrode obtained at 0.2 and 5 A g^−1^ are 2435 and 1280 mAh g^−1^, respectively. This superior performance (Figure 6c) is associated with the relatively low *R*_ct_ (Figure S9a, Supporting Information) and high *D*_Li_^+^ (Table 2) among the electrodes.

Figure 6d shows the cycling stability data of the electrodes measured at 1.0 A g^−1^. After 300 charge–discharge cycles, the Si/NO‐0.5 h, Si/NO‐1 h, Si/NO‐2 h, and Si/NO/C‐N electrodes retained 54%, 77%, 70%, and 90% of their initial capacities, respectively. The impedance evolution of the electrodes after cycling was also investigated (see Figure S9b, Supporting Information). As shown in Figure 6e, the increase in *R*_ct_ for Si/NO/C‐N is greatly suppressed compared with those for the other electrodes. SEM images of various electrodes before and after cycling are compared in Figure S12 (Supporting Information). The morphology of the Si/NO‐0.5 h electrode was distorted after cycling. The Si particles clearly expanded and agglomerated. Moreover, the electrode surface was covered by a thick SEI layer, which can increase the electrode impedance and lead to marked capacity deterioration. In contrast, with the double nitrogenation layer, the structure of the Si/NO/C‐N electrode was highly preserved after cycling, which explains its exceptional cyclability. Figure S13 (Supporting Information) shows cross‐sectional SEM images of the Si/NO‐0.5 h and Si/NO/C‐N electrodes after 50 charge–discharge cycles. Much less volume expansion and better integrity of the latter electrode are confirmed. As shown in Table S4 (Supporting Information), the cycling stability of the Si/NO/C‐N electrode is among the best reported for Si‐based anodes. Under harsh conditions (a capacity of >1900 mAh g^−1^ and a rate of 1.0 A g^−1^), where substantial and rapid electrode volume variation occurred, satisfactory cyclability was still achieved for the Si/NO/C‐N electrode, which showed a steady CE of 99.9% up to 300 cycles. Note that we did not optimize the binder and electrolyte recipes and did not use any sophisticated electrode architectures to maximize the cycle life. If these strategies are adopted, further improvement in electrode cycling stability can be expected.

**Figure 7:**
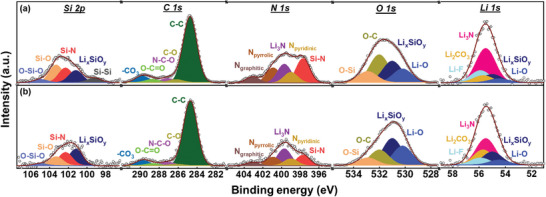
XPS spectra of Si/NO/C‐N electrodes a) after two conditioning cycles and b) after 300 charge–discharge cycles.

**Figure** **7**a,b compares the XPS data of the Si/NO/C‐N electrodes after two conditioning cycles and after 300 charge‐discharge cycles. As shown in the Si 2p data, the Si─O and Si─N species are consumed and Li*_x_* SiO*_y_* (at 101.2 eV) emerges after conditioning (compared with the data of the as‐prepared electrode shown in Figure 5d). The N 1s spectrum indicates the clear formation of Li_3_N (at 399.7 eV),^[^ ^32^, ^64^, ^65^ ^]^ which is a highly favorable SEI component as it provides high mechanical robustness, great passivation ability, and high Li^+^ conductivity.^[^ ^36^, ^66^ ^]^ Besides Li*_x_* SiO*_y_* and Li_3_N, LiF, Li_2_CO_3_, and Li─O species are constituents of the SEI (according to the Li 1s, C 1s, and O 1s spectra). As shown, after 300 charge–discharge cycles, the XPS characteristics are close to those of the electrode that underwent only conditioning cycles. This indicates that the SEI layer on the Si/NO/C‐N electrode is robust and steady, which is in line with the observed great electrode cyclability.

**Figure 8:**
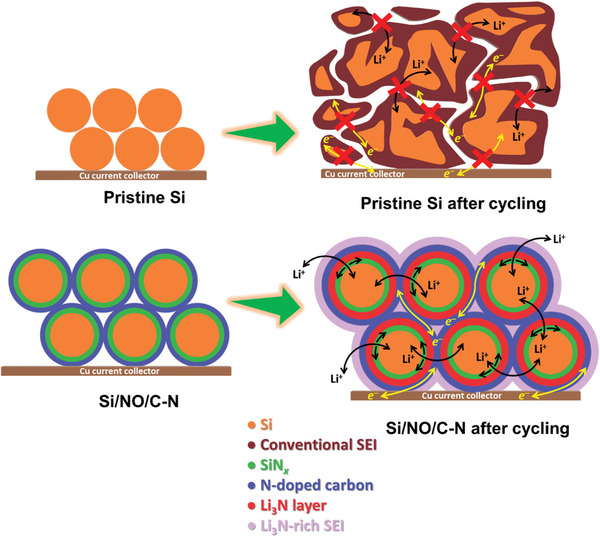
Schematic illustration of structure evolution for pristine Si and Si/NO/C‐N electrodes upon charge–discharge cycling.

**Figure** **8** shows the structural evolution of the pristine Si and Si/NO/C‐N electrodes upon cycling. The former electrode has low electronic conductivity and loose interparticle connectivity. The uncontrolled volume variation of the Si particles during lithiation/delithiation causes serious mechanical degradation. The repeated breakdown and reformation of the SEI lead to SEI accumulation and thus a thickness increase, which isolates the Si particles and hinders Li^+^ and *e* ^−^ transport, resulting in rapid electrode performance deterioration. In contrast, the Si/NO/C‐N particles are covered by an inner SiN*_x_* layer and an outer N‐doped carbon layer, which provide good electronic conduction, ensuring a close connection between the active material particles and promoting electrolyte wettability (see Figure S14, Supporting Information). Upon charging/discharging, percolation conduction pathways formed within the electrode. The produced Li_3_N (converted from SiN*_x_*) is responsible for the high Li^+^ conductivity and great passivation ability,^[^ ^33^ ^]^ which lead to high charge–discharge performance. The multiple amorphous nitrogenation layers (SiN*_x_*/Li_3_N/N‐doped carbon), which are protective and flexible, buffer the Si volume change and wrap the particles to prevent their pulverization. Consequently, the electrode interface is stabilized and good cyclability is achieved.

**Figure 9:**
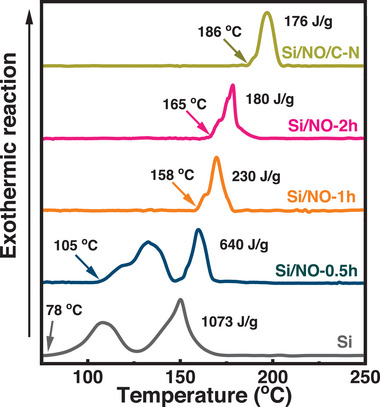
DSC data of various lithiated electrodes conducted under N_2_ gas with a heating rate of 5 °C min^−1^.

To assess the safety properties, the interfacial exothermic reactions between various lithiated electrodes and the electrolyte were examined using DSC. **Figure** **9** shows the obtained data for the pristine Si, Si/NO‐0.5 h, Si/NO‐1 h, Si/NO‐2 h, and Si/NO/C‐N samples after two conditioning cycles and in the lithiation state. The pristine Si showed an exothermic reaction starting at ≈78 °C, which is attributed to the breakdown of the SEI layer.^[^ ^67^ ^]^ Afterward, multiple exothermic reactions, which included the thermal decomposition of the electrolyte and direct interaction between the highly active, lithiated Li*_x_* Si with the electrolyte,^[^ ^68^, ^69^, ^70^ ^]^ occurred, leading to a second peak at ≈150 °C. The total heat released was as high as ≈1073 J g^−1^. For Si/NO‐0.5 h, the exothermic onset temperature increased to ≈105 °C. However, probably due to the incomplete coverage of Li_3_N (i.e., the SEI composition is not uniform), the first exothermic peak related to SEI became split. As shown, increasing the NO treatment time increases the exothermic onset temperature and reduces the total heat generated. Of note, the exothermic peak associated with SEI was significantly diminished when the NO treatment time was 1 h or longer. In addition, the Li_3_N‐rich layer seems to alleviate the interaction between the lithiated electrode and the electrolyte, decreasing the second exothermic peak intensity. As shown in Figure 9, adding the N‐doped carbon coating on the Si/NO‐1 h particles further enhanced thermal stability. The measured exothermic onset temperature and the total heat generated for the Si/NO/C‐N sample are ≈186 °C and ≈176 J g^−1^, respectively. This is the first work to study the thermal properties of N‐doped Si anodes and show that the formed Li_3_N‐based SEI can greatly promote electrode safety.

**Figure 10:**
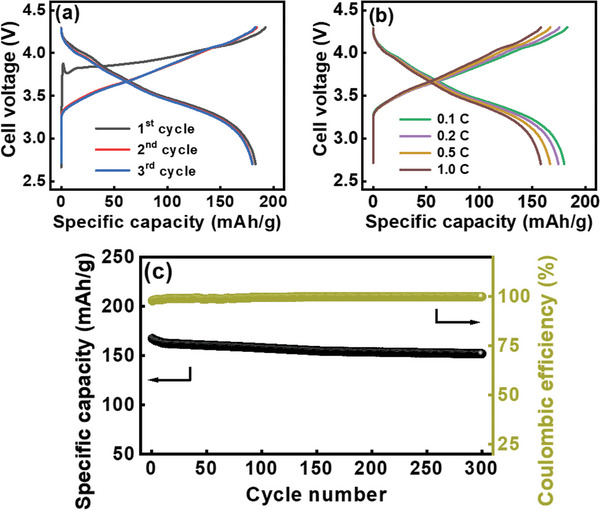
a) Initial charge–discharge curves measured at 0.1 C and b) charge–discharge profiles measured at various rates of Si/NO/C‐N||LiNi_0.8_Co_0.1_Mn_0.1_O_2_ full cell. c) Cycling stability of Si/NO/C‐N||LiNi_0.8_Co_0.1_Mn_0.1_O_2_ full cell measured at 0.5 C for 300 cycles.

To evaluate the potential of the proposed anode for practical applications, an Si/NO/C‐N||LiNi_0.8_Co_0.1_Mn_0.1_O_2_ full cell was constructed with an anode‐to‐cathode capacity ratio of 1.15. **Figure** **10**a,b shows the charge–discharge profiles measured during conditioning cycles at 0.1 C and during operation at various C rates (1 C = 200 mA g^−1^ for LiNi_0.8_Co_0.1_Mn_0.1_O_2_). As shown, the reversible specific capacities (based on both the anode and cathode materials) of the cell are 180, 175, 167, and 158 mAh g^−1^ at 0.1, 0.2, 0.5, and 1 C, respectively. The gravimetric energy density of this cell calculated based on the discharge profile at 0.1 C is approximately 585 Wh kg^−1^, where the mass does not include the electrolyte, current collectors, binders, conductive agents, and separator. This promising energy density indicates the merit of the Si/NO/C‐N anode. Figure 10c shows the cycling stability of the cell measured at 0.5 C. The Si/NO/C‐N||LiNi_0.8_Co_0.1_Mn_0.1_O_2_ cell retains ≈90% of its initial capacity after 300 charge–discharge cycles. The Si particles with a double nitrogenation layer formed using NO gas have great cyclability for high‐energy‐density LIB applications.

## 3 Conclusion

Urea, melamine, and NO were, for the first time, confirmed to be valid precursors for nitrogenizing Si materials. Among them, NO is particularly interesting. NO at a concentration of only 1500 ppm in the carrier gas (at 850 °C) is sufficient for modifying the materials and altering their electrochemical performance. We found that bare Si particles are more suitable than carbon‐coated Si particles as a raw material for the nitrogenation reaction using NO for the optimization of Li^+^ storage properties because the carbon layer blocks the interaction between NO and the Si cores. Accordingly, the rate capability, *R*_ct_, *D*_Li_^+^, and cycling stability of the Si/NO series of electrodes are superior to those of the Si/C‐N‐NO electrode. However, the NO treatment time of the Si particles should be appropriately controlled. Over‐nitrogenation led to excessive formation of Li_3_N during cycling, increasing both the Li^+^ transport distance across the SEI and electrode resistance. The Si/NO/C‐N sample with a double nitrogenation layer (an inner SiN*_x_* layer and an outer N‐doped carbon layer) had high electronic and Li^+^ conduction between particles; the layers promoted electrolyte wettability and provided good protection for the Si cores (alleviating pulverization and uncontrolled SEI accumulation). An excellent initial CE of 89% and high capacities of 2435 and 1280 mAh g^−1^ at 0.2 and 5 A g^−1^, respectively, were obtained. The constructed Si/NO/C‐N||LiNi_0.8_Co_0.1_Mn_0.1_O_2_ full cell showed a high energy density of 585 Wh kg^−1^ and retained ≈90% of its initial capacity after 300 charge–discharge cycles. Of note, according to the DSC data, the formed N‐containing SEI was thermally robust and mitigated the interaction between the lithiated electrode and the electrolyte. The measured exothermic onset temperature and total heat generated for the Si/NO/C‐N electrode are ≈186 °C and ≈176 J g^−1^, respectively, which are much better than those (≈78 °C and ≈1073 J g^−1^) for the pristine Si electrode. The proposed NO nitrogenation method is facile, effective, and scalable. The obtained Si/NO/C‐N anode material with a double nitrogenation layer has great potential for high‐energy‐density, long‐life, and high‐safety LIB applications.

## 4 Experimental Section

### Nitrogenation of Carbon‐Coated Si and Bare Si Particles

Micrometer‐size Si powder (*D*_50_: 1.8 µm; purity >99.9%) was provided by Super Energy Material Inc., Taiwan. Planetary ball milling was performed for 24 h to reduce the *D*_50_ value of the Si particles to ≈100 nm (the obtained sample is denoted as pristine Si). The nitrogenation of the carbon‐coated Si particles was performed using one of two methods. In the wet chemical method, an ethanol solution containing glucose and a nitrogen precursor (either melamine or urea) was prepared. The Si powder, glucose, and nitrogen precursor (melamine or urea) were mixed in a weight ratio of 6:3:1. The mixture was stirred for 5 h and the resulting material was filtered, dried, and then calcined at 850 °C under Ar for 5 h. The obtained samples are denoted as Si/C‐N‐M and Si/C‐N‐U, respectively. In the gas reaction method, an Si/C sample was first prepared using the wet chemical procedure above but without any nitrogen precursor. Then, it was heated at 850 °C for 5 h under an NO‐containing flow, which consisted of 3000 ppm NO/He gas (50 cc min^−1^) and 10% H_2_/Ar gas (50 cc min^−1^). This sample is denoted as Si/C‐N‐NO.

The nitrogenation of bare Si powder was conducted by heating the pristine Si at 850 °C under the same NO‐containing flow for 0.5, 1, or 2 h. The resulting samples are denoted as Si/NO‐0.5 h, Si/NO‐1 h, and Si/NO‐2 h, respectively. The Si/NO‐1 h sample was further subjected to glucose loading and 5 h of NO heating at 850 °C to create a double nitrogenation layer. This sample is denoted as Si/NO/C‐N.

### Preparation of Electrolyte, Electrodes, and Cells

Battery‐grade LiPF_6_, ethylene carbonate (EC), diethyl carbonate (DEC), and fluoroethylene carbonate (FEC) were purchased from Kishida Chemical Co., Ltd. The solvents were treated using molecular sieves to remove residual water before use. The LiPF_6_ (1 M) was dissolved in EC/DEC mixed solvent (1:1 by volume) with 5 wt% FEC additive to form the electrolyte. The electrode slurry was made of 80 wt% active material powder, 10 wt% conducting Super P, and 10 wt% sodium polyacrylate binder in deionized water. The slurry was cast onto Cu foil using a doctor blade and then vacuum‐dried for 8 h at 100 °C. The obtained electrodes were punched to fit the required dimensions of a CR2032 coin cell. The amount of active material loading was ≈2 mg cm^−2^. The thickness values of all electrodes were approximately 60 µm. Li foil and a glass fiber membrane were used as the counter electrode and separator, respectively, in a half‐cell configuration. For full‐cell construction, the developed negative electrode was paired with a LiNi_0.8_Mn_0.1_Co_0.1_O_2_ positive electrode with a capacity ratio of 1.15:1. The negative electrode was prelithiated to 10% capacity in a half cell prior to the full‐cell assembly. The coin cells were assembled in an Ar‐filled glove box (Vigor Tech. Co. Ltd.), where the moisture and oxygen content levels were maintained at below 0.2 ppm.

### Material and Electrochemical Characterizations

XRD (Bruker D2 Phaser) was used to determine the crystal structures of the samples. SEM (JEOL JSM7800F Prime) and TEM (JEOL F2100F) were used to study the morphology and microstructures. The particle size distribution was measured using DLS (Otsuka ELSZ‐2000), with ethanol used as the dispersant. The Raman spectra were collected using a spectrometer (LabRAM HR 800) with an excitation laser wavelength of 633 nm. TGA (TA Instruments Q500) was conducted under air with a heating rate of 5 °C min^−1^. XPS (Thermo Fisher Scientific ESCALAB Xi^+^) was employed to examine the surface chemistry of the samples. The X‐ray source was Al K_α_ radiation (1486.6 eV). The contact angles between various electrodes and 1 M LiPF_6_ EC/DEC/FEC electrolyte were measured using a digital goniometer (Sindatek Model 100SB). DSC (NETZSCH 3500) was conducted under N_2_ gas with a heating rate of 5 °C min^−1^. The cycled electrodes were removed from the coin cells and rinsed in the glove box with the EC/DEC solvent. The electrodes were then transferred to the XPS chamber using an air‐tight vessel. The EIS measurements were conducted using a potential perturbation amplitude of 10 mV within a frequency range of 10^6^–10^−2^ Hz (with a BioLogic VSP‐300 potentiostat). A battery tester (NEWARE CT‐4000) was used to study the charge–discharge properties, such as capacity, CE, rate capability, and cycling stability, of various cells at 25 °C. The GITT was used to assess the apparent Li^+^ diffusion coefficients (*D*_Li_^+^) of various electrodes.

### Statistical Analysis

The CV, EIS, GITT, and charge–discharge measurements of various electrodes were repeated at least three times to ensure validity. The data deviation was typically within ≈3%. The reported values are the medians. All the XPS spectra were calibrated with the binding energy of the C 1s peak (284.7 eV). The data fitting was done using XPSPEAK 4.1 software. For XRD data, the background subtraction and phase identification were conducted using the EVA and TOPAS programs provided in the Bruker software package. Origin software was used for data analysis and processing.

## Conflict of Interest

The authors declare no conflict of interest.

## Supporting information

Supporting Information

## Data Availability

The data that support the findings of this study are available from the corresponding author upon reasonable request.
