# Supplementary material for: Double Nitrogenation Layer Formed Using Nitric Oxide for Enhancing Li+ Storage Performance, Cycling Stability, and Safety of Si Electrodes
Source: Adv Sci (Weinh). 2024 Apr 24;11(25):2310062. doi: 10.1002/advs.202310062 (PMC11220681; doi:10.1002/advs.202310062)
Supplement: Supplementary file 1 — Supporting Information [file ADVS-11-2310062-s001.pdf]

ADVANCED SCIENCE
Open Access

Supporting Information

for *Adv. Sci.*, DOI 10.1002/advs.202310062

Double Nitrogenation Layer Formed Using Nitric Oxide for Enhancing Li^+ Storage Performance, Cycling Stability, and Safety of Si Electrodes

*Rahmandhika Firdauzha Hary Hernandha, Bharath Umesh, Jagabandhu Patra, Chung-Jen Tseng, Chien-Te Hsieh, Ju Li and Jeng-Kuei Chang**

Supporting Information

Double Nitrogenation Layer Formed Using Nitric Oxide for Enhancing Li^+ Storage Performance, Cycling Stability, and Safety of Si Electrodes

*Rahmandhika Firdauzha Hary Hernandha, Bharath Umesh, Jagabandhu Patra, Chung-Jen Tseng, Chien-Te Hsieh, Ju Li, and Jeng-Kuei Chang**

Table S1. Tap density of various samples.

Sample	Tap density (g cm^{-3})
Si	0.28
Si/C	0.57
Si/C-N-M	0.64
Si/C-N-U	0.67
Si/C-N-NO	0.69
Si/NO-0.5h	0.72
Si/NO-1h	0.79
Si/NO-2h	0.80
Si/NO/C-N	0.84

Table S2. Elemental composition of pristine Si, Si/C, Si/C-N-M, Si/C-N-U, Si/C-N-NO, Si/NO-0.5h, Si/NO-1h, Si/NO-2h, and Si/NO/C-N samples measured using XPS.

Sample	Si (at%)	C (at%)	N (at%)	O (at%)
Si	68.2	7.0	0.0	24.8
Si/C	22.6	43.1	0.0	34.3
Si/C-N-M	22.1	41.3	3.7	32.9
Si/C-N-U	21.6	43.1	3.9	31.4
Si/C-N-NO	26.9	28.7	3.8	40.6
Si/NO-0.5h	30.7	15.4	2.5	51.4
Si/NO-1h	29.5	11.4	4.4	54.7
Si/NO-2h	28.0	11.1	5.0	55.9
Si/NO/C-N	19.8	30.1	4.1	46.0

Table S3. Electronic conductivity values of pristine Si, Si/C, Si/C-N-M, Si/C-N-U, Si/C-N-NO, Si/NO-0.5h, Si/NO-1h, Si/NO-2h, and Si/NO/C-N samples.

Sample	Electronic conductivity ($\times 10^{-1}$ S cm^{-1})
Si	1.35
Si/C	3.21
Si/C-N-M	4.02
Si/C-N-U	4.18
Si/C-N-NO	4.63
Si/NO-0.5h	2.88
Si/NO-1h	3.19
Si/NO-2h	3.21
Si/NO/C-N	5.02

Table S4. Performance comparison of Si/NO/C-N with various N-doped Si-based anodes reported in the literature.

No.	Material	Synthesis method	Initial reversible capacity (mAh g^{-1})	Initial Coulombic efficiency (%)	Capacity retention (%) / number of cycle / current rate (A g^{-1})	Reference
**Nitrogenation on Si**						
1	α-Si_3N_4	Ball milling (reduce size with time dependent)	83	~40	N/A	*Mater. Lett.* 2003, 57, 3063–3069
2	α-Si_3N_4w + 30 % Si composites	Ball milling	>800	~40	~27% / 50 / 0.08	*Solid State Ion.* 2007, 178, 1107–1112
3	a-Si_3N_4p + 30 % Si composites	Ball milling	~1000	~50	~47% / 50 / 0.08	*Solid State Ion.* 2007, 178, 1107–1112
4	$Si_{3-x}M_xN_4$ (M=Fe)	Manual mix of α-Si_3N_4+Fe and pelletized	470.6	50.5	45.4% / 50 / 0.1 C	*Int. J. Electrochem. Sci.* 2007, 2, 478–487
5	Binder-free a-SiN/BCNT	Hot filament CVD & sputtering	607	N/A	62% / 10 / 2 x 10^{-4} A cm^{-2}	*Electrochim. Acta* 2010, 55, 2269–2274
6	a-$SiN_{0.92}$	Pulsed Laser Deposition	1800	~75	72.2% / 100 / 0.2 C	*J. Power Sources* 2013, 231, 186–189
7	a-SiN_x/graphene	CVD, dispersion, and heat treatment	>3000	70	45.2% / 200 / 0.5	*J. Mater. Chem. A* 2014, 2, 14577–14584
8	N1-Si/CMAs (a-$SiN_{0.73}$)	Two-step DC sputtering on Cu Micro-cone arrays (CMAs)	2789	80	40% / 200 / 0.2 C	*J Power Sources* 2016, 325, 64–70
9	a-SiN_x-H	Plasma Enhanced CVD	32	69.1	N/A (capacity was	*Electrochim. Acta* 2018,

					increasing until 200th and decreasin g until 350th cycle)	268, 241–247
10	$Si@Si_3N_4@C$	Two-step gas–solid reaction	3093.8	91.5	81.3% / 200 / 0.5	*Energy Storage Mater.* 2020, 24, 565–573
11	$SiN_{0.7}$ nanoparticles	Gas-phase synthesis	1212	~55	82% / 300 / 0.5 C	*Part. Part. Syst. Charact.* 2021, 38, 2100007
12	p-Si@SiN	Direct nitriding hydrogenated porous Si	2584	81	84% / 200 / 0.5	*J. Energy Chem.* 2022, 69, 616–625
**Nitrogenation on Carbon Coating Layers**						
13	N-doped graphene-like nanosheets on Si	Ball milling and heat treatment	1323	82.2	61.9% / 50 / 0.2	*J. Mater. Chem. A* 2014, 2, 11254–11260
14	CN@P-Si	Wet chemical mixing using reflux method and carbonization	~3500	84	82% / 100 / 0.8	*Electrochim. Acta* 2016, 209, 299–307
15	NG/Si@NC	Wet chemical mixing and carbonization	1498	76	82% / 100 / 0.5	*Electrochim. Acta* 2016, 192, 22-29
16	Core-shell Si/N-doped C	*In situ* interfacial polymerization and heat treatment	1861	~61	88% / 50 / 0.2	*Funct. Mater. Lett.* 2016, 9, 1650055
17	Si@NC-NPs	Polymerization and heat treatment	~975	~65	90.7% / 300 / 1 C	*Curr. Appl. Phys.* 2017, 17, 1087-1093
18	Si@N-C	Aerosol-assisted assembly & physisorption process	2172	~60	~66% / 200 / 1	*ACS Energy Lett.* 2017, 2, 1279−1287
19	Si@NPC	*In situ* trimerization of p-	1412	57.1	>100% / 200 / 1	*Nanoscale* 2017, 9, 8871–8878

		benzenedinitrile in molten $ZnCl_2$ & annealing				
20	OM-Si@NC	Polymerization and heat treatment	~1819	71.4	73.5% / 200 / 1	*ACS Appl. Mater. Interfaces* 2017, 9, 32829−32839
21	Si–NC	Solid-state reaction method	997.8	70.7	88.6% / 50 / 0.1	*Funct. Mater. Lett.* 2018, 11, 1850067
22	N-C@m-Si	Hydrothermal method and carbonization	~1230	~58	~44% / 50 / 0.1 C	*Sci. Rep.* 2019, 9, 3318
23	Si/NG	Carbonization and subsequent low-temperature chemical reduction	2127	80	64.2% / 240 / 1	*Chem. Commun.* 2019, 55, 2644-2647
24	3D Si/NGC	Magnesiothermic reduction reaction from ZIF-67	1200	56	85.5% / 300 / 0.2	*J. Alloys Compd.* 2019, 777, 190-197
25	Si@void@NC	Simple solution coating and high-temperature pyrolysis	~1100	74.6	70% / 400 / 0.2	*ACS Appl. Energy Mater.* 2022, 5, 1471-1477
26	3D NPC@Si	High voltage sputtering	~2100	84	~75% / 100 / 0.5	Chem. Eng.J. 2018, 343, 78-85
**Nitrogenation on Both Si and Carbon Coating**						
	**Si/NO/C-N**	**NO treatment**	**2471**	**89**	**90% / 300 / 1**	**This work**

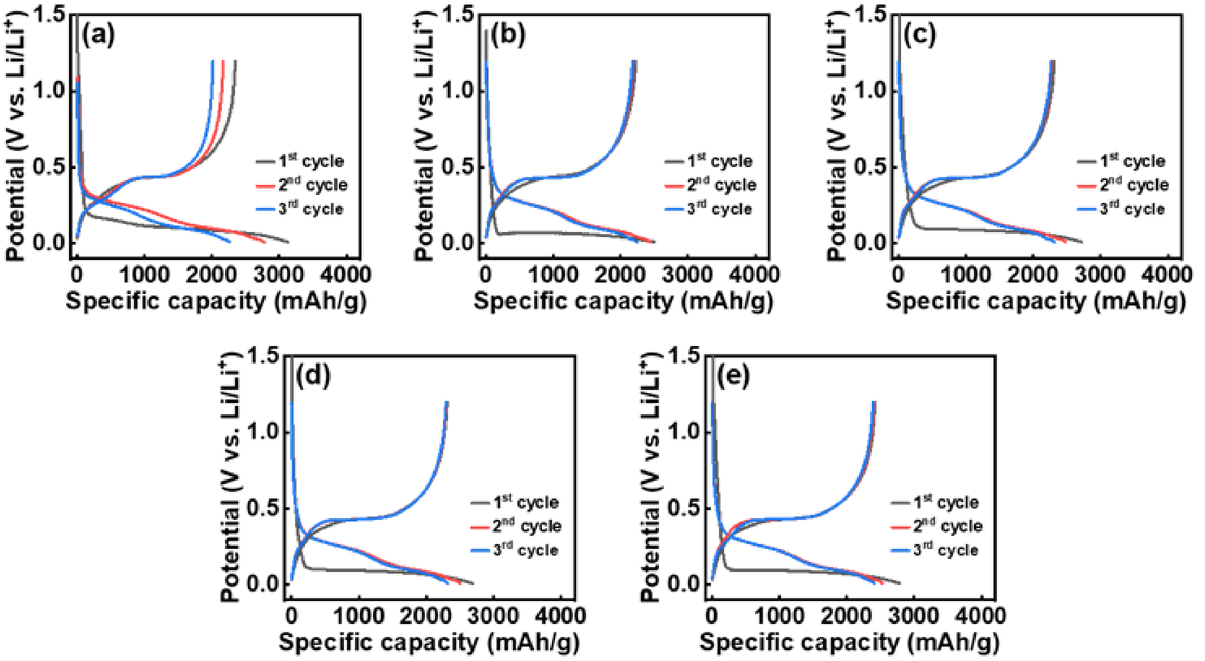

Figure S1. Initial three charge-discharge cycles of (a) Si, (b) Si/C, (c) Si/C/N-M, (d) Si/C/N-U, and (e) Si/C/N-NO electrodes measured at 0.2 A g^{-1}.

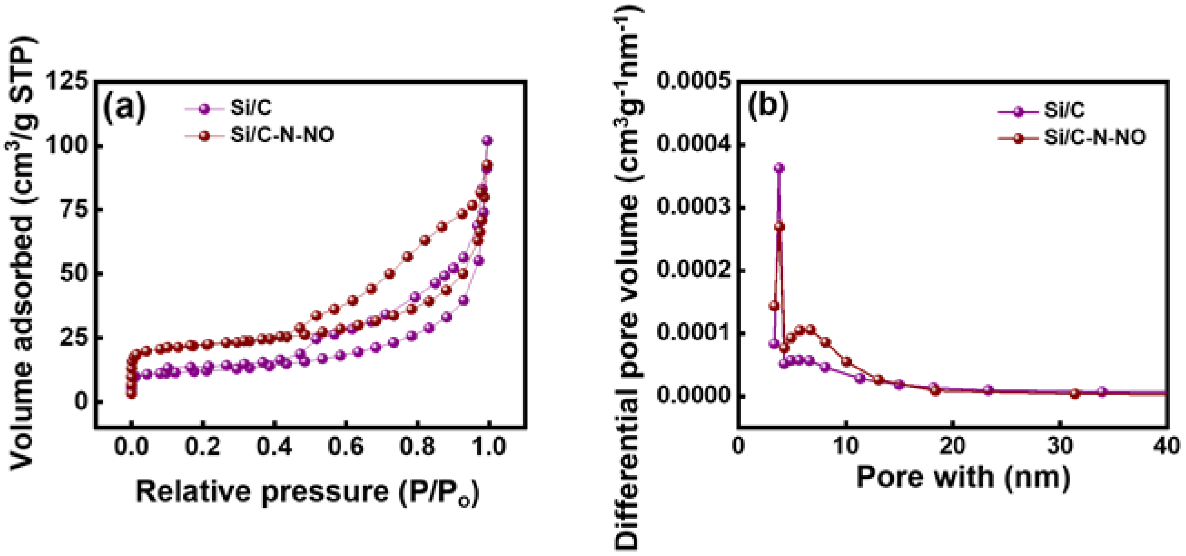

Figure S2. (a) N_2 adsorption/desorption isotherms and (b) pore size distribution curves of Si/C and Si/C-N-NO samples.

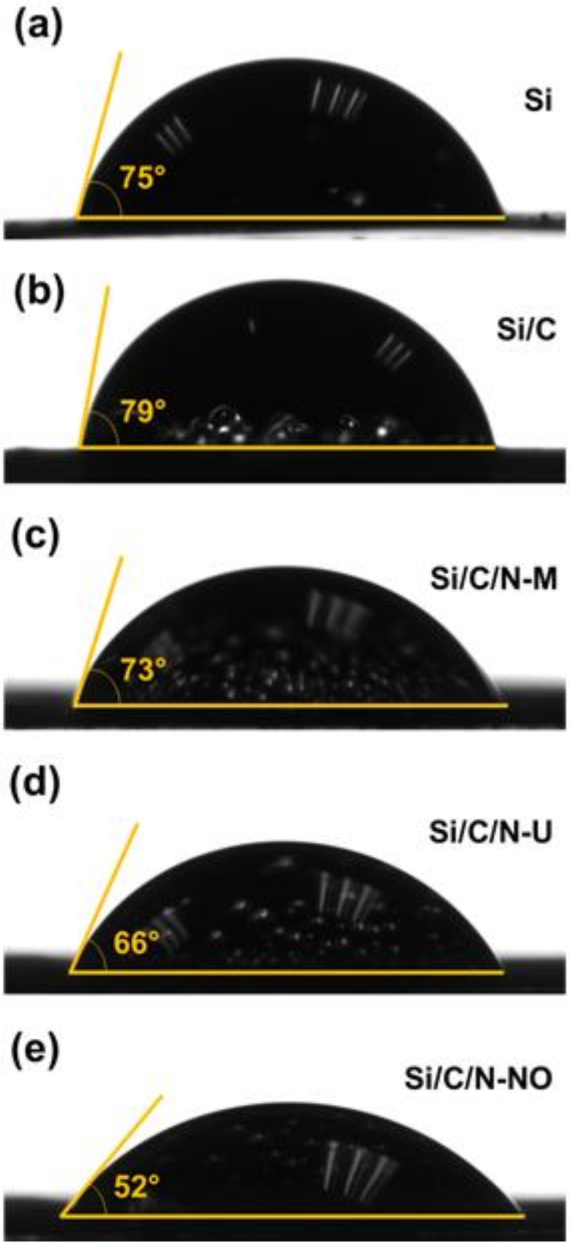

Figure S3. Contact angle measurements of (a) pristine Si, (b) Si/C, (c) Si/C/N-M, (d) Si/C/N-U, and (e) Si/C/N-NO electrodes toward electrolyte.

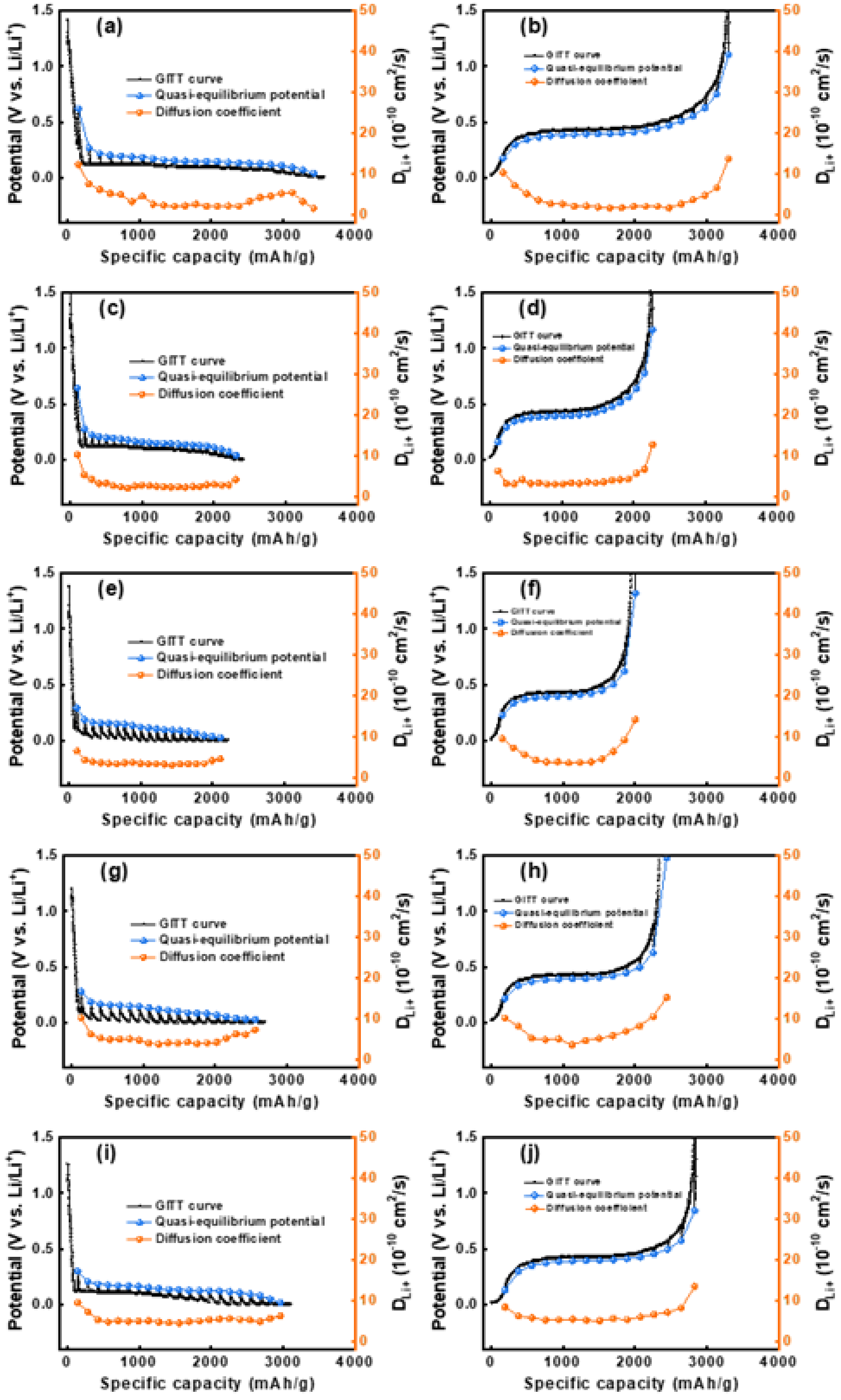

Figure S4. GITT data for (a,b) pristine Si, (c,d) Si/C, (e,f) Si/C/N-M, (g,h) Si/C/N-U, and (i,j) Si/C/N-NO electrodes measured during lithiation and delithiation processes. Please see the note below for the $D_{Li}{}^{+}$ calculation.

Note: The apparent Li^+ diffusion coefficients (D_{Li^+}) of the electrodes can be calculated based on the GITT. The following equation is used to evaluate the D_{Li^+} values.

$$D_{Li+} = \frac{4}{\pi\tau}\left(\frac{mV}{MS}\right)^2\left(\frac{\Delta E_S}{\Delta E_\tau}\right)^2$$

In the equation, m is the mass of the electroactive material, and M (28.08 g mol^{-1}) and V (12.06 cm^3 mol^{-1}) are the molar mass and molar volume of Si, respectively. τ represents the time for which the constant current pulse is applied (600 minutes). ΔE_S and ΔE_τ denote the change in the steady-state voltage (E_0) of the cell for each applied galvanostatic current pulse and the total transient voltage change of the cell during the step, respectively. S stands for the geometric surface area of the electrode (i.e., 1.33 cm^2).

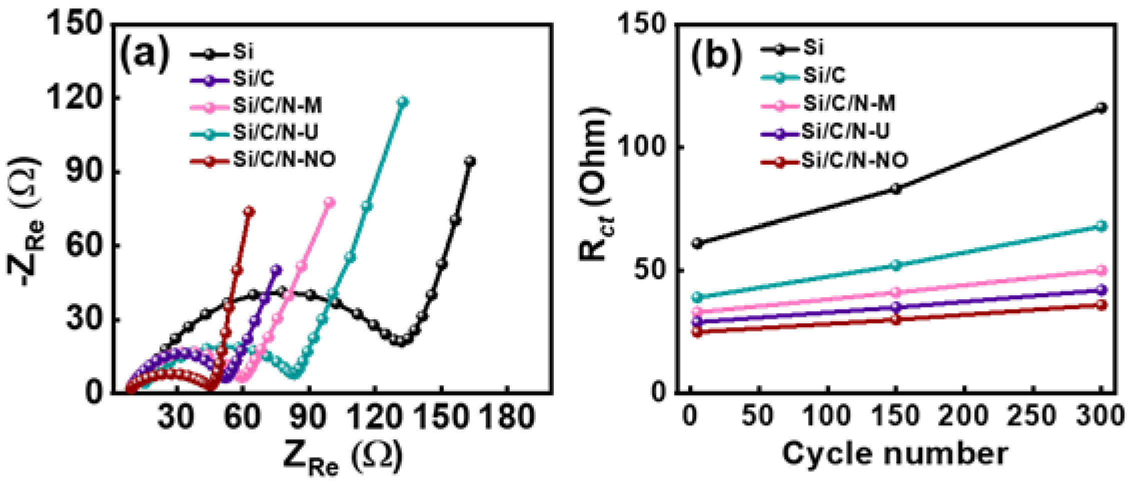

Figure S5. (a) EIS spectra of various electrodes acquired after 300 charge-discharge cycles. (b) Variation of R_{ct} values for various electrodes with respect to charge-discharge cycle number.

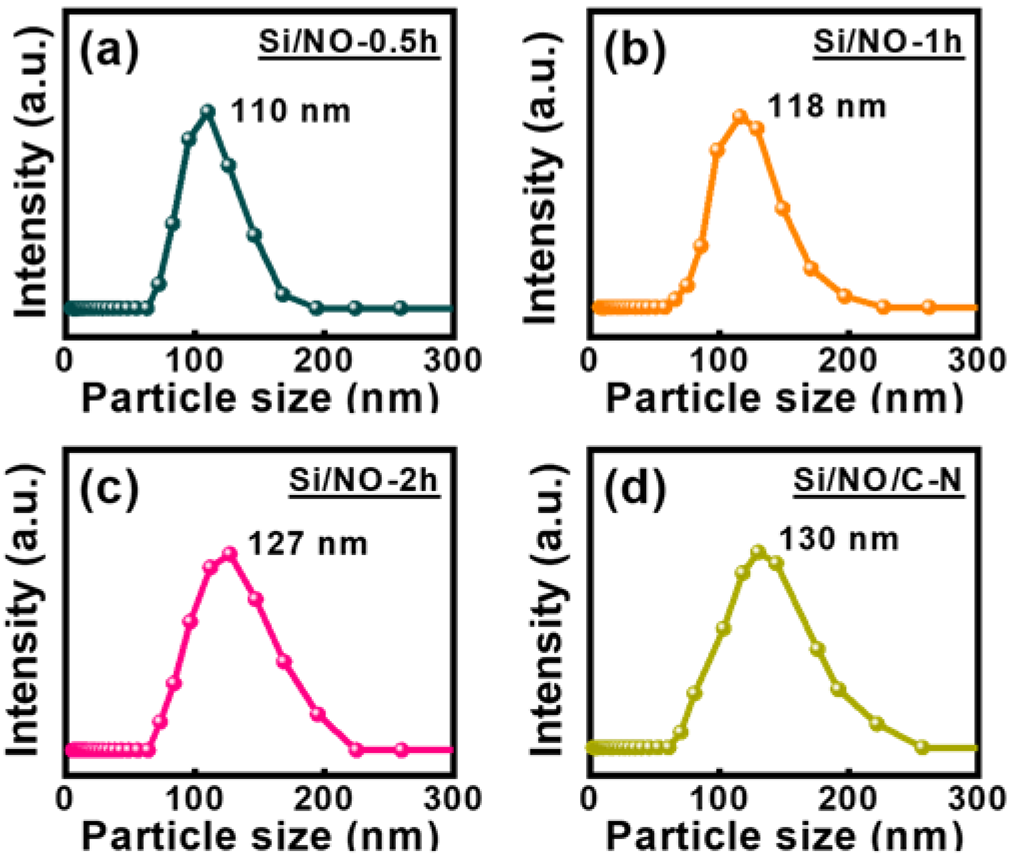

Figure S6. DLS daata for (a) Si/NO-0.5h, (b) Si/NO-1h, (c) Si/NO-2h, and (d) Si/NO/C-N samples.

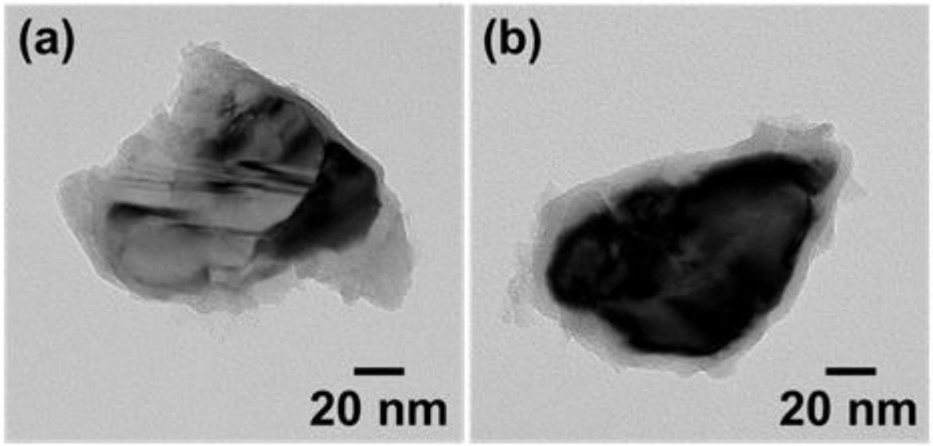

Figure S7. Low-resolution TEM images of (a) Si/NO-1h and (b) Si/NO/C-N samples.

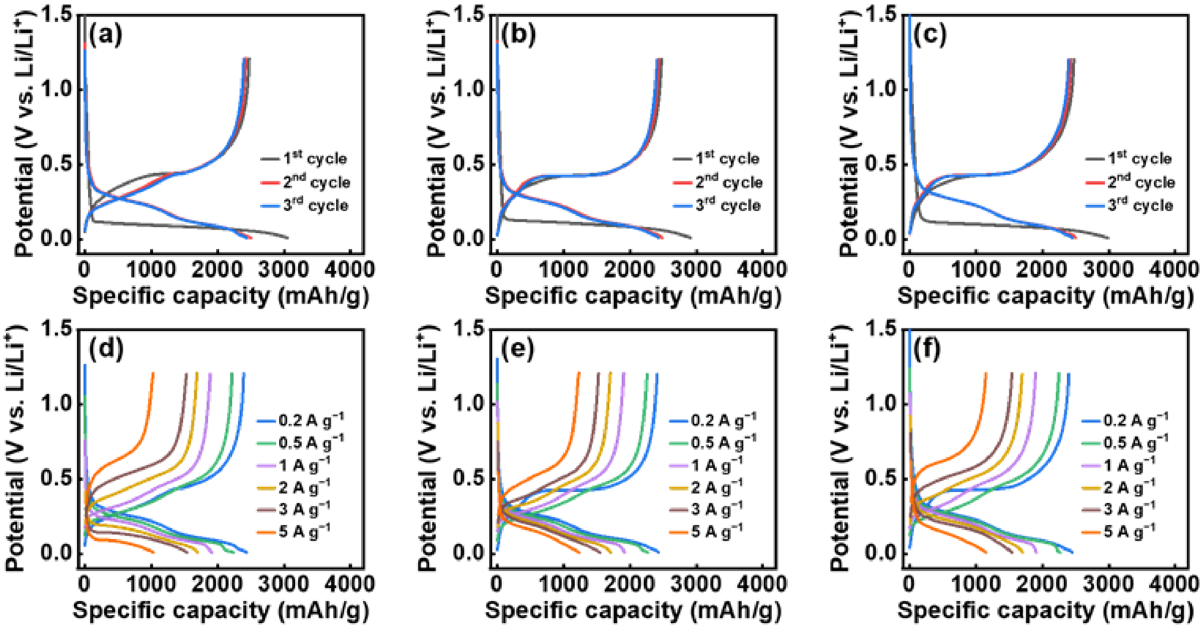

Figure S8. Initial three conditioning cycles performed at 0.2 A g^{-1} and charge-discharge profiles measured at various rates for (a,d) Si/NO-0.5h, (b,e) Si/NO-1h, and (c,f) Si/NO-2h electrodes.

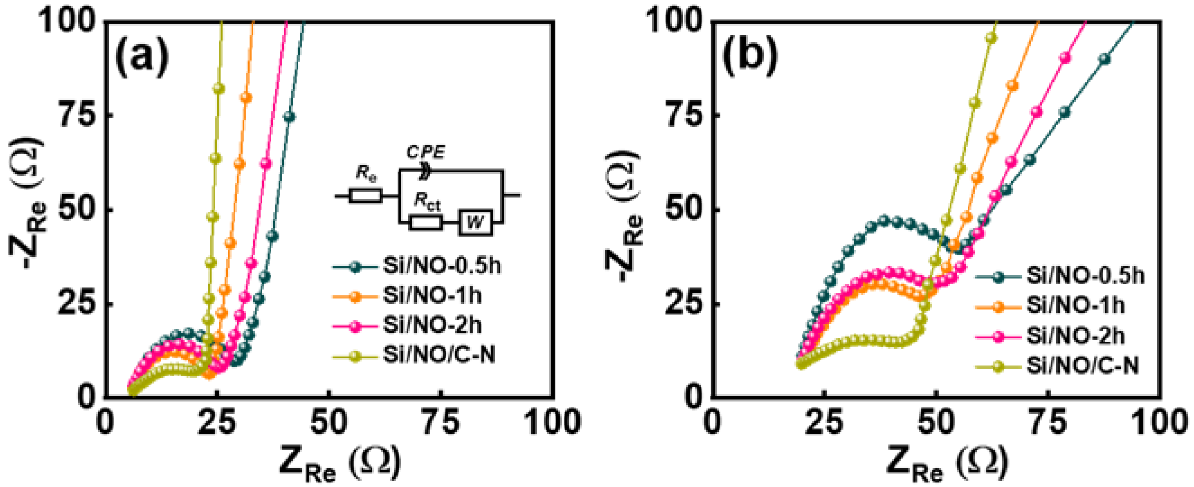

Figure S9. EIS spectra acquired after (a) conditioning cycles and (b) after 300 charge-discharge cycles for Si/NO-0.5h, Si/NO-1h, Si/NO-2h, and Si/NO/C-N electrodes.

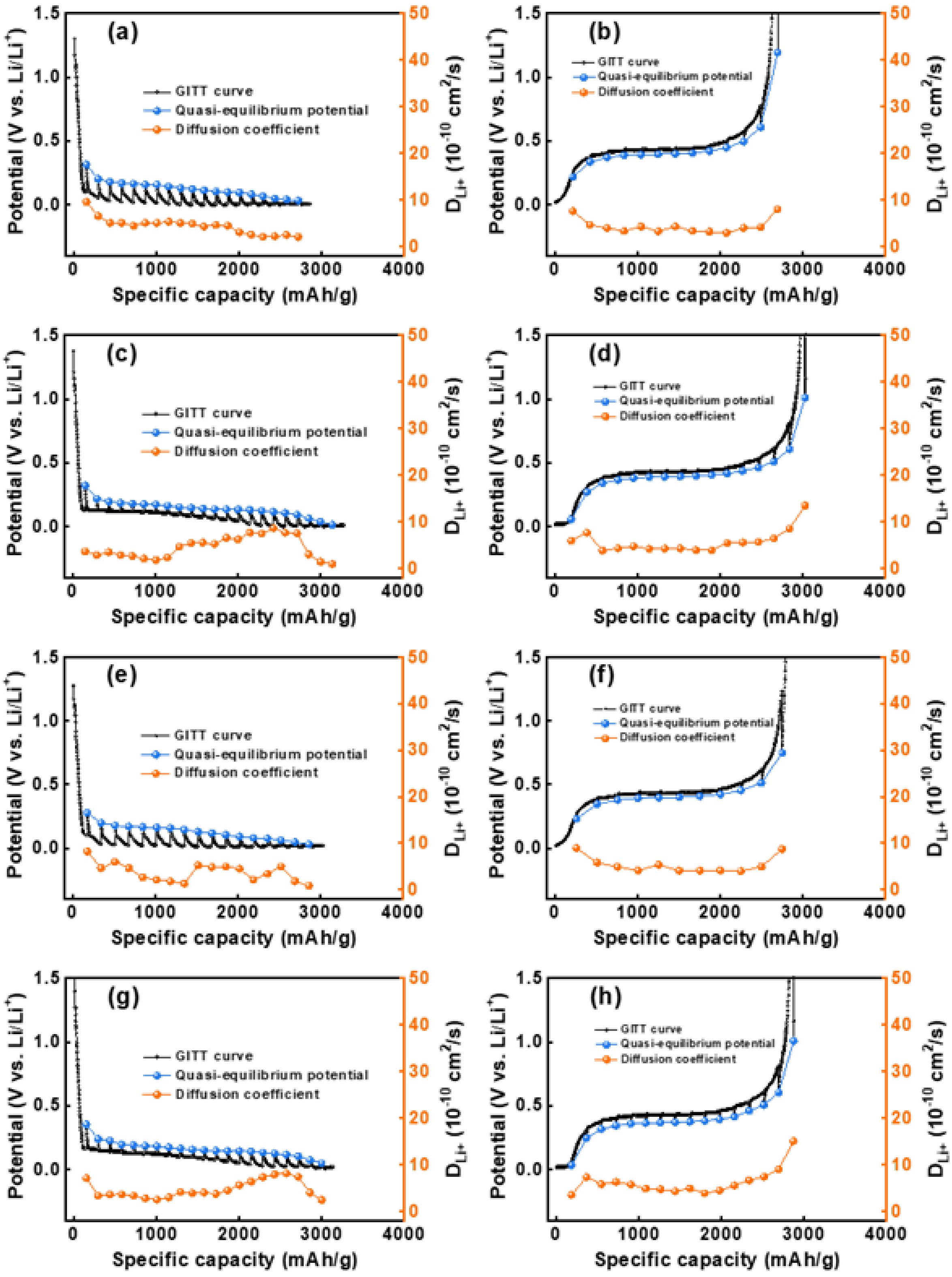

Figure S10. GITT data for (a,b) Si/NO-0.5h, (c,d) Si/NO-1h, (e,f) Si/NO-2h, and (g,h) Si/NO/C-N electrodes measured during lithiation and delithiation processes.

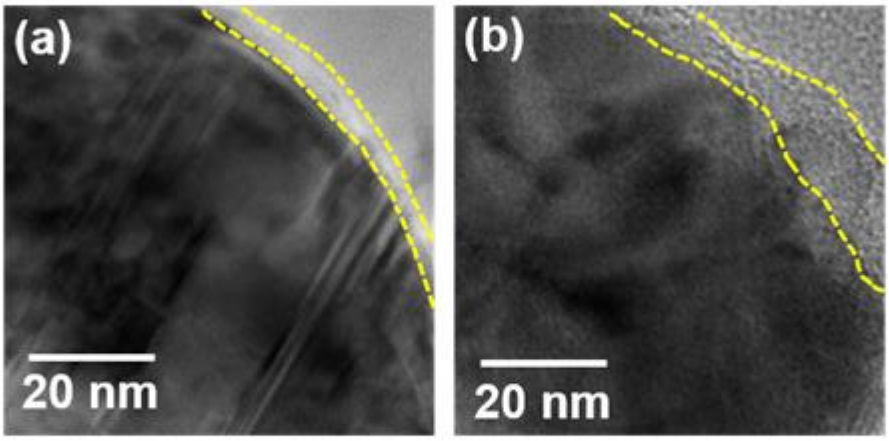

Figure S11. High-resolution TEM images for (a) Si/NO-1h and (b) Si/NO-2h samples after two conditioning cycles.

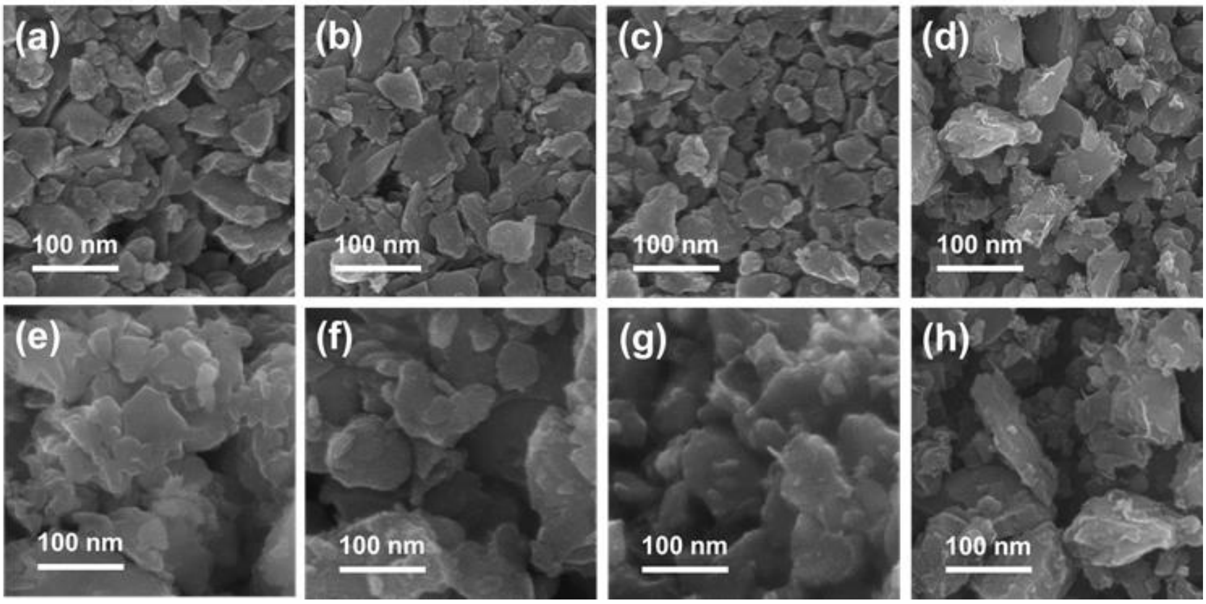

Figure S12. SEM images of (a,e) Si/NO-0.5h, (b,f) Si/NO-1h, (c,g) Si/NO-2h, and (d,h) Si/NO/C-N electrodes. (a)−(e) are pristine electrodes and (e)−(h) are the electrodes after 300 charge-discharge cycles.

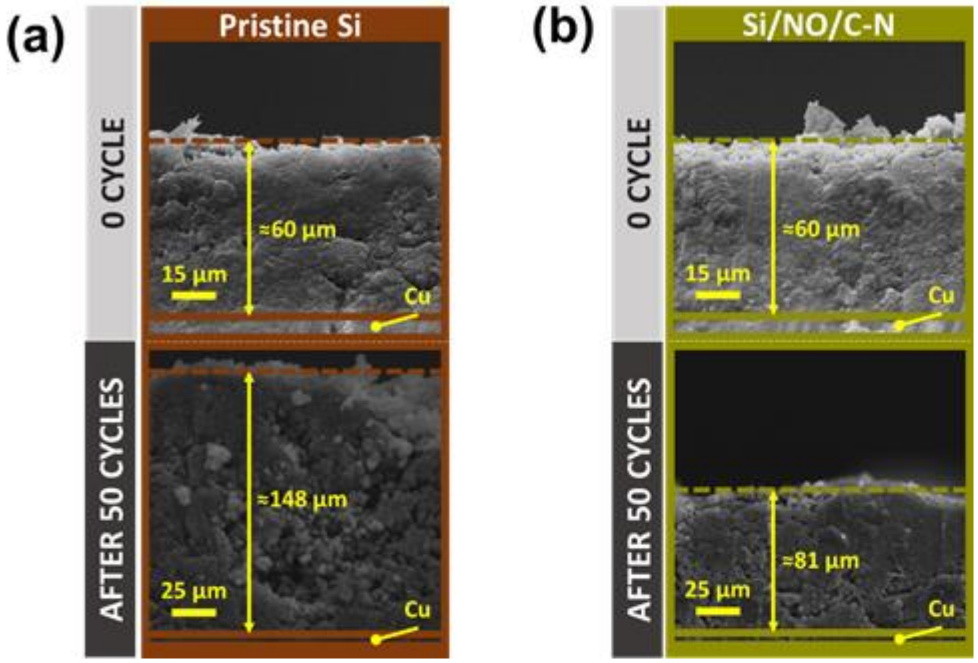

Figure S13. Cross-sectional SEM images of (a) Si/NO-0.5h and (b) Si/NO/C-N electrodes after 50 charge-discharge cycles.

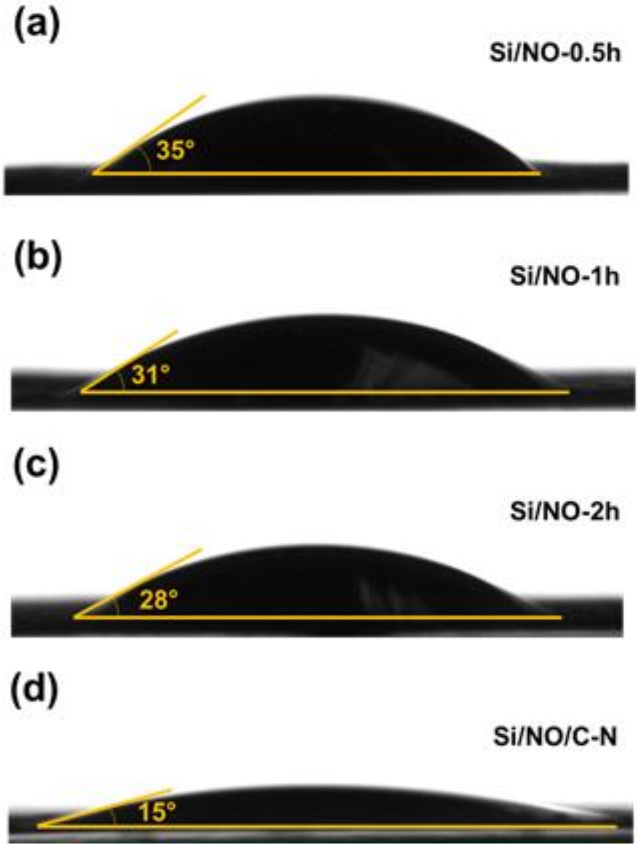

Figure S14. Contact angle measurements of (a) Si/NO-0.5h, (b) Si/NO-1h, (c) Si/NO-2h, and (d) Si/NO/C-N electrodes toward electrolyte.
